# TVET programme and health-related quality of life among low-income populations during the COVID-19 pandemic in Malaysia

**DOI:** 10.3389/fpubh.2024.1164056

**Published:** 2024-03-05

**Authors:** Ruhizan Mohammad Yasin, Maw Pin Tan, Mas Ayu Said, Mohd Sattar Rasul, Nithiah Thangiah, Hussein Rizal, Amirah Shazana Magli, Muslimah Ithnin, Hazreen Abdul Majid, Rozmi Ismail, Tin Tin Su

**Affiliations:** ^1^STEM Enculturation Research Center, Universiti Kebangsaan Malaysia, Bangi, Malaysia; ^2^Department of Medicine, Faculty of Medicine, University of Malaya, Kuala Lumpur, Malaysia; ^3^Centre for Epidemiology and Evidence-Based Practice, Department of Social and Preventive Medicine, Faculty of Medicine, University of Malaya, Kuala Lumpur, Malaysia; ^4^Department of Social and Preventive Medicine, Faculty of Medicine, Centre for Population Health, University of Malaya, Kuala Lumpur, Malaysia; ^5^Universiti Malaya, Kuala Lumpur, Malaysia; ^6^School of Health Sciences, KPJ Healthcare University, Nilai, Malaysia; ^7^Department of Nutrition, Faculty of Public Health, Universitas Airlangga, Surabaya, Indonesia; ^8^School of Rehabilitation Sciences, AECC University College, Bournemouth, United Kingdom; ^9^Psychology and Human Wellbeing Research Centre, Faculty of Social Sciences and Humanities, Universiti Kebangsaan Malaysia, Bangi, Malaysia; ^10^South East Asia Community Observatory (SEACO) and Global Public Health, Jeffrey Cheah School of Medicine and Health Sciences, Monash University Malaysia, Bandar Sunway, Malaysia

**Keywords:** HRQOL, Malaysia, low-income, education, skill, TVET

## Abstract

**Introduction:**

Education improves the economy and quality of life. The availability of skilled education in Malaysia is not restricted to the younger generation but is available to people of all ages, including those with low incomes.

**Methods:**

This study used the EuroQol 5-Dimension 5-Level (EQ- 5D-5L) tool during the COVID-19 pandemic to examine relationships between socio-demographics, knowledge, and attitudes towards education and outcomes of health-related quality of life (HRQOL). Between September and October 2020 and January and February 2021, a cross-sectional study using a multi-stage sampling technique was carried out.

**Results:**

A total of 1,997 adults participated, with a mean age of 45.17 (SD 14.113). In total, 74.9% had good knowledge, while 59.8% had a positive attitude towards skill education. In univariate analyses, the EQ-5D-5L score was related to age, income, education level, marital status, employment status, financial strain level, and knowledge and attitude towards skilled education. Generalised linear model analyses demonstrated that lower EQ-5D-5L scores were associated with older age, financial constraints, and a negative attitude towards skills education. However, additional adjustments for knowledge and attitude towards skills education show only an increase in age and financial strain was significant.

**Conclusion:**

The findings suggest that appropriate strategies be implemented to increase low-income populations’ knowledge and attitude towards skill education. Improving education may improve the quality of life for this vulnerable group. Additionally, a qualitative study can be conducted to determine the barriers to low-income households participating in skilled education to fill in the knowledge gap.

## 1 Introduction

In its 1946 assembly, the World Health Organisation (WHO) included quality of life in its adopted definition of health, which is “a state of complete physical, mental, and social well-being and not merely the absence of disease or infirmity” (1). When the health status of a population needs to be known, the WHO recognises the health-related quality of life (HRQOL) as a measurement of an individual’s health status in public health (2). The primary goal of the HRQOL measurement was to improve and protect people’s and communities’ health (3). HRQOL has been used in cross-sectional studies in the community and populations with specific diseases to help researchers and policymakers identify the main predictors of quality of life (QOL) (4–6). Based on these studies, interventions to enhance the quality of life of the populations studied were then developed and evaluated.

Previous studies on the impact of the economic crisis have shown that vulnerable groups, including those with low income, lower education, older adult, and single parents, experienced a greater reduction in quality of life than the general population (7–9). The Department of Statistics of Malaysia (DOSM) categorised Malaysians into three income groups, namely, the Top 20% (T20), the Middle 40% (M40), and the Bottom 40% (B40) (10). During the COVID-19 pandemic, the B40 group was involved mainly in low-skilled economic activities, had exceedingly more labour, and was not suitable to work from home. As a result, B40 households risk losing their jobs and sources of income (11). According to a report by DOSM, in the B40 group, the incidence of absolute poverty increased from 5.6% in 2019 to 8.4% in 2020. Meanwhile, hardcore poverty is estimated to increase from 0.4% in 2019 to 1.0% in 2020 (12). Compared to the other groups, the B40 group faced greater challenges regarding food insecurity, financial strain, employment issues, and increasing mental health problems (13–15).

According to the United Nations Educational, Scientific, and Cultural Organisation (UNESCO), the goals of the Strategy for Technical and Vocational Education and Training (TVET), 2016–2021, which supported Member States such as Malaysia, were to improve the relevance of their TVET systems and to equip all youth and adults with the skills needed for employment, decent work, entrepreneurship, and lifelong learning, as well as to contribute to the implementation of the 2030 Agenda for Sustainable Development (16). It is also generally regarded as a skill education programme comparable to other countries (17, 18). TVET has been widely recognised in numerous countries and unions due to its technical reliance on capacity building, achieving higher income. It has decreased poverty, particularly among low-income populations (17–19).

A study by Oesch (20) reported that unemployment disproportionately affects low-skilled workers in Organisation for Economic Co-operation and Development (OECD) countries. Thus, a quality and flexible skills education, including TVET, will allow adults in low-income groups to raise their earnings inequality and increase their economy (21, 22). Additionally, increasing their skills will benefit the participants’ stability, working participation, and, eventually, their health and wellbeing (23, 24). Despite the acknowledgement by UNESCO of the importance of TVET, studies on populations’ knowledge and attitudes towards TVET, including in Malaysia, remain low. In addition, studies looking at the impact of these factors on HRQoL are also very scanty, either in Malaysia or globally.

Previous research found that socio-demographic factors influence poor health status (6, 25). Simultaneously, knowledge is the foundation for determining attitudes, intentions, and behaviour. Research using theories such as the theory of planned behaviour (TPB), the health belief model (HBM), and the knowledge–attitude–practice/behaviour (KAP) model demonstrated a relationship between knowledge factors influencing practice or behaviour among communities (26–28). Therefore, this study employs the dimensions of (1) socio-demographic, (2) knowledge towards skill education, and (3) attitude towards skill education with HRQOL outcome.

Given the importance of this low-income population study and the current government policy on addressing inequalities and promoting the low-income population to attain skilled education to increase their income (29), this study investigates the demographic factors and knowledge and attitude towards skill education associated with HRQOL. Our hypotheses were that (1) socio-demographic factors were correlated to HRQoL and (2) good knowledge and positive attitude towards skill education were positively related to HRQoL in low-income populations. Exploring the knowledge and attitude of the population towards skill education or TVET could provide specifically targeted intervention strategies to Malaysians, particularly low-income groups. Meanwhile, the HRQOL will provide a picture of their health status and relate it with their knowledge and attitude towards skill education or TVET.

## 2 Methods

A cross-sectional survey of low-income Malaysians was used in this study. Participants with low income were classified as being in the Bottom 40 (B40) group, defined as having an monthly income of less than 4,850 Ringgit Malaysia (equal to 1,089 USD) (10). Participants in the B40 income group were identified using household income data.

### 2.1 Sampling, recruitment, and data collection

With a population totalling 32.75 million as of the first quarter of 2021 (12), the study enlisted 2,125 participants from six distinct Malaysian states, namely, Selangor, Pahang, Sabah, Sarawak, Pulau Pinang, and Johor. The states represents each region, based on simple random sampling (12). Probability proportionate to sample size was used to calculate the sample needed from each state. To illustrate, Selangor represented the central region in the Western Peninsula, Pulau Pinang covered the northern region, Johor represented the southern region, and Pahang represented the eastern region. Meanwhile, Sabah and Sarawak were designated as the Eastern Peninsula.

The sampling frame comprised heads of households (typically males) aged 18 years and older falling within the B40 income group, as determined through household income data obtained from the DOSM in 2020 (12). The identification of municipalities within each respective state involved the use of random selection procedures. Specifically, the sampled municipalities included Seberang Perai, Selayang, Ampang, Subang Jaya, Shah Alam, Petaling Jaya, Johor Bahru, Kuantan, Kuching Utara, Kuching Selatan, Padawan, and Kota Kinabalu. Subsequently, a simple random sampling method was employed to select participants aged 18 years old from B40 households within each municipality. In cases where the data collector identified discrepancies, typically through verbal confirmation, signifying that a household deviated from the B40 classification recorded by DOSM, the corresponding participant from that household would then be excluded. For instance, participants from households with a monthly income exceeding 4,850 Ringgit Malaysia or those below 18 years of age.

Surveys were carried out with individual households aged 18 years and older through self-administered questionnaires. Due to movement restrictions imposed during the COVID-19 pandemic, data were collected for two intervals: 1 September 2020 to 24 October 2020 and 25 January 2021 to 15 February 2021. Throughout the interview process, data collectors adhered to standard operating procedures (SOP), including practices such as physical distancing, wearing face masks, and regularly using hand sanitisers. Additionally, the MySejahtera application was utilised to record and assess the COVID-19 risk for both data collectors and participants. Field activity approval was obtained from the Faculty of Medicine and Institute for Advanced Learning, University Malaya, in compliance with SOP guidelines set by the Ministry of Health, Malaysia.

### 2.2 Ethics statement

This study complies with the ethics upheld by the Helsinki Declaration with the criteria for studies reviewed by an accredited University of Malaya Research Ethics Committee (UMREC). Before taking the survey, the participants were given a consent form explained to them by the researcher. All of the participants were over the age of 18 and provided written informed consent.

### 2.3 Survey measures

Survey items consisted of demographic questions and quantitative measures of knowledge towards skills education, attitude towards skills education, and health-related quality-of-life instruments.

The measure for knowledge of skill education and attitude towards skills education were developed by the researchers based on the qualitative survey done earlier in phase 1. These two measures are all perception measures. The qualitative survey was done using focus group interview (FGI) with the representative of the households (parents). The items developed represent the themes from the FGI. This approach helps ensure that the questionnaire items are grounded in the experiences and perspectives of the target population. This procedure of developing the questionnaire item was suggested by Creswell & Creswell (30) and Denzin and Lincoln (31). The items were then validated by four research experts to review the proposed content of the questionnaire for face validity. Then, it was piloted with 47 individuals to conduct the reliability analysis to obtain Cronbach’s alpha value.

#### 2.3.1 Demographics factors

Demographic items included questions regarding sex, marital status, ethnicity, education level, employment status during COVID-19, household income category, having a skills certificate and financial strain. Mutually exclusive racial categories were formed, with the main racial groups in Malaysia being Malay, Chinese, and Indian. Indigenous Peoples, Pribumi Sabah and Pribumi Sarawak, were categorised into the Pribumi group (12). The education level is divided into no school/preschool, primary, secondary, tertiary, or other categories. Other education is for those who did not receive formal education provided by the Malaysian government.

Among the B40 participants, their income was classified based on household income classification by DOSM (12) were B1, less than RM2,500 (equal to 562 USD), B2 from RM2,501 (equal to 562 USD) to RM3,170 (equal to 712 USD), B3 from RM3,171 (equal to 712 USD) to RM3,970 (equal to 891 USD), and B4 from RM3,971 (equal to 891 USD) until RM4,850 (equal to 1,089 USD).

The participants were asked whether they had experienced five situations that indicated their financial strain status, which are (i) insufficient money, (ii) sufficient for basic needs only, (iii) sufficient for most necessities, (iv) sufficient to purchase goods and services but unable to make savings, or (v) sufficient to purchase goods and services and capable to make savings. Based on the Malaysian Financial Planning Council (32), these statements have been adapted to suit the Malaysian context. The “Sufficient for most necessities”, “Sufficient to purchase goods and services but unable to make savings”, or “Sufficient to purchase goods and services and capable of making savings” categories were collapsed into “sufficient to buy all necessities”.

#### 2.3.2 Knowledge of skill education factors

Measures of knowledge towards skill education assessed participants’ knowledge of the skill or training education provided by the government, industry, or academy. First, four research experts reviewed the proposed content of the questionnaire for face validity. Then, it was piloted with 47 individuals to conduct the reliability analysis to obtain Cronbach’s alpha value. Four items were used in which participants indicated the frequency of (1) knowledge in finding opportunities to improve knowledge/education, (2) knowledge of assistance from the government to improve education and training, (3) knowledge in seeking educational opportunities and assistance from agencies other than government (e.g., NGOs & Industry), and (4) know what educational facilities to improve skills provided by the government. Using a 4-point rating scale (1 = Strongly disagree to 4 = Strongly agree), participants indicated the frequency of each item. Cronbach’s alpha of these four items was 0.882.

#### 2.3.3 Attitude towards skill education factors

Six items were used to measure attitude towards skill education among participants, which comprised (1) did not continue studies due to financial problems, (2) believing that there is always a solution to any challenge, (3) having negative feelings and emotional instability due to the COVID-19 pandemic, (4) believe it is essential to look for opportunities to improve job skills, (5) always curious about nearby skills accreditation centres, and (6) believe that after the Covid-19 pandemic, there is a need to improve vocational skills to help the family. Participants responded to each item on a 4-point Likert scale, with 1 indicating strongly disagree and 4 indicating strongly agree. Two items measuring the frequency were reverse coded. Cronbach’s alpha of these six items was 0.888.

#### 2.3.4 Health-related quality-of-life outcomes

The EuroQol 5-Dimension 5-Level (EQ-5D-5L) tool, which has been translated and validated in Malay, is a descriptive system that divides health into five distinct dimensions: (1) mobility, (2) self-care, (3) usual activities, (4) pain/discomfort, and (5) anxiety/depression (33, 34). Within each dimension, participants were asked to describe their current health status on a 5-point (Likert scale) severity scale, which included the following: (1) no problems, (2) minor problems, (3) moderate problems, (4) severe problems, and (5) unable or extreme problems. The EQ-5D-5L instrument has been validated in the general population and patient groups, paving the way for its use in Malaysian health research (35, 36). The information gathered is displayed as an index value. The Malaysian EQ-5D value set was used to calculate values for all health states, ranging from −0.442 to 1 (36, 37).

### 2.4 Data analysis

The descriptive analyses in this study were used to examine the participant characteristics. Frequency analysis was used to identify missing and extreme values. Responses were summed for a range of 4 to 16 for the knowledge towards skills education factor. The knowledge was classified using the median score. Those with total scores higher than the median value were classified as having good knowledge. Simultaneously, responses for the attitude towards skills education factor were summed for a possible range of 6 to 24. Those with total scores higher than the median value were classified as having a positive attitude towards skills education.

The EQ-5D-5L values were the primary outcome of this study. The EQ-5D-5L were not normally distributed (*p* < 0.05) after a normality test using the Shapiro–Wilk test. Thus, Spearman’s correlation and the chi-square test were used to determine the relationship between independent variables with EQ-5D-5L. Variables with a *p* < 0.250 value were then included in the multivariate analysis (38). A two-sided *p*-value of 0.05 was used to determine statistical significance. For this study, IBM SPSS Statistics 23 was used.

## 3 Results

### 3.1 Demographic characteristics

The total sampled population was 2,125. Due to missing data, we only analysed and included a sample of 1,997 households. Hence, the response rate is 94.0%. The participants consisted of 1,346 (67.4%) males and 650 (32.6%) females, with a mean age of 45.17 (SD 14.113). The study conducted interviews with the heads of households, who were predominantly males. In instances where the designated head of the household was unavailable, the interview was conducted with the wife or an adult child in their stead. Nearly two-thirds (70.5%) of the participants were married, whereas 14.8, 12.6, and 2.1% were single, widows, and separated or divorced, respectively. More than half (69.5%) of participants were Malay. Table 1 presents the demographic characteristics and the association between demographic characteristics and EQ-5D-5L scores.

**Table 1:** Association of EQ-5D-5L scores with demographics characteristics of participants (*n* = 1,997).

Variables	*n* (%)	Mean (SD)	*p*-value
*Gender*			0.006*
Male	1,346 (67.4)	0.938 (0.155)	
Female	650 (32.6)	0.931 (0.133)	
*Marital status*			0.001*
Single	295 (14.8)	0.951 (0.128)	
Married	1,405 (70.5)	0.940 (0.146)	
Widow	250 (12.6)	0.899 (0.156)	
Separated/divorce	42 (2.1)	0.897 (0.197)	
*Ethnicity*			0.050
Malay	1,383 (69.5)	0.941 (0.140)	
Chinese	172 (8.6)	0.894 (0.215)	
Indian	164 (8.2)	0.939 (0.146)	
Pribumi	175 (8.8)	0.930 (0.131)	
Others	95 (4.8)	0.945 (0.112)	
*Education level*			0.001*
No education/preschool	105 (5.4)	0.904 (0.175)	
Primary	167 (8.6)	0.906 (0.170)	
Secondary	1,145 (58.9)	0.937 (0.151)	
Tertiary	506 (26.0)	0.956 (0.160)	
Others	20 (1.0)	0.854 (0.238)	
*Employment status during COVID-19*			0.001*
Yes	1,407 (70.5)	0.948 (0.140)	
No	589 (29.5)	0.905 (0.166)	
*Household income category*			0.004*
<RM2500	1,390 (69.6)	0.928 (0.160)	
RM2,501–3,170	243 (12.2)	0.954 (0.122)	
RM3,171–3,970	145 (7.3)	0.936 (0.147)	
RM3,971–4,850	219 (11.0)	0.961 (0.939)	
*Have a skills certificate*			0.706
Yes	150 (7.5)	0.940 (0.140)	
No	1847 (92.5)	0.935 (0.150)	
*Financial strain*			0.002*
Not enough	241 (12.1)	0.892 (0.222)	
Enough for basic needs	1,257 (62.9)	0.939 (0.135)	
Enough to buy all the necessities	499 (25.0)	0.944 (0.136)	
*Knowledge of skills education category*			0.057
Poor (<11)	475 (24.1)	0.930 (0.150)	
Good (12–16)	1,495 (74.9)	0.940 (0.140)	
*Attitudes towards skills education category*			0.057
Negative (<16)	784 (40.2)	0.930 (0.150)	
Positive (17–23)	1,168 (59.8)	0.940 (0.130)	
EQ-5D-5L total index		0.935 (0.150)	

Over half (58.9%) had a secondary education level, and more than half (69.6%) were in the B1 income category with a household income of less than RM2,500 per month. Nearly two-thirds (70.5%) of the participants were still employed during the COVID-19 pandemic. From the open-ended responses, only 7.5% of participants had skills certificates. Among the skills attained were automotive, culinary, sewing, computer, and electrical and construction certifications. For financial strain, during the survey conducted, 62.9% had enough money to buy basic needs, 25.0% had enough money to buy all the necessities, and 12.1% did not have enough money even to buy basic needs.

### 3.2 Knowledge and attitude toward skill education

The mean knowledge and attitude towards skill education were 11.98 (SD 4.10) and 16.23 (SD 3.92), respectively. Nearly two-thirds (74.9%) had good knowledge, while more than half (59.8%) had a positive attitude towards skill education (Table 1).

### 3.3 HRQOL

The participants scored an overall mean EQ-5D index score of 0.935 (SD 0.150). Spearman’s rank correlation was computed to assess the relationship between age, household income, knowledge, and attitude with EQ-5D-5L scores. Spearman correlation results showed that age had a weak negative correlation and household income had a weak positive correlation with EQ-5D-5L scores (*p* < 0.001). While knowledge and attitude had a weak positive correlation with EQ-5D-5L scores (*p* < 0.005). For categorical variables, there was a significant difference in EQ-5D-5L scores across sex, marital status category, employment status, household income category, ad financial strain status (*p* < 0.005) (Table 1).

### 3.4 Multivariate linear regression analysis

Table 2 summarises the multivariate linear regression analysis to determine the relative contributions of individual factors on HRQOL. Within the unadjusted model, age, employment status, education, income, financial strain, knowledge, and attitude towards skills education was associated with differences in quality of life. In the first adjusted model, age (β = − 0.02, 95% CI = − 0.02, − 0.01), financial strain (β = − 0.046, 95% CI = − 0.068, − 0.024), and attitude towards skills education (β = − 0.013, 95% CI = − 0.025, − 0.001) were significantly associated with HRQOL. The mean of HRQOL was found to be 0.046 units lower in those with financial strain compared to those with sufficient financial capabilities. In contrast, those with a negative attitude towards skills education had 0.013 units lower in the mean of HRQOL compared to those with a positive attitude towards skills education. Those with financial constraints and a negative attitude towards skills education suffer from a lower quality of life. However, following additional adjustment for knowledge towards skills education and attitude towards skills education were no longer significantly associated with HRQOL. At the same time, age (β = − 0.02, 95% CI = − 0.02, − 0.01) and financial strain (β = − 0.046, 95% CI = − 0.067, − 0.024) remained significant. This suggests that knowledge of education accounted for the relationship between attitude towards education and HRQOL.

**Table 2:** Multiple linear regression analyses for socio-demographic and education predictors with HRQOL.

Variable	Unadjusted			Adjusted Model 1			Adjusted Model 2		
	B	SE	95% CI (LL, UL)	B	SE	95% CI (LL, UL)	B	SE	95% CI (LL, UL)
Age (year)	**−0.002**	0.0002	−0.002, −0.001	**−0.002**	0.0003	−0.002, −0.001	**−0.002**	0.0003	−0.002, −0.001
*Gender*									
Male	0.009	0.007	−0.004, 0.022	0.006	0.007	−0.008, 0.020	0.006	0.007	−0.008, 0.020
Female	Ref			Ref			Ref		
*Ethnicity*									
Malaysia	−0.001	0.014	−0.028, 0.027	−0.007	0.014	−0.035, 0.021	−0.007	0.014	−0.036, 0.021
Chinese	−0.020	0.017	−0.054, 0.014	−0.005	0.018	−0.039, 0.030	−0.005	0.018	−0.039, 0.030
Indian	−0.001	0.017	−0.035, 0.033	0.003	0.017	−0.031, 0.037	0.002	0.017	−0.032, 0.036
Pribumi	−0.017	0.017	0.051, 0.018	−0.023	0.017	−0.056, 0.011	−0.023	0.017	−0.057, 0.011
Others	Ref			Ref			Ref		
*Marital status*									
Single	0.025	0.023	−0.020, 0.070	−0.017	0.023	−0.062, 0.029	−0.017	0.023	−0.062, 0.029
Married	0.009	0.022	−0.034, 0.051	−0.004	0.022	−0.047, 0.039	−0.005	0.022	−0.047, 0.038
Widowed	−0.029	0.023	−0.074, 0.016	−0.016	0.023	−0.061, 0.029	−0.017	0.023	−0.061, 0.028
Separated/divorced	Ref			Ref			Ref		
*Employment status*									
Employed	**0.035**	0.007	0.022, 0.048	0.008	0.007	−0.006, 0.023	0.008	0.007	−0.007, 0.022
Unemployed	Ref			Ref			Ref		
*Education level*									
Primary	0.002	0.032	−0.062, 0.065	0.008	0.032	−0.055, 0.072	0.008	0.032	−0.055, 0.072
Secondary	0.013	0.032	−0.049, 0.074	0.012	0.031	−0.049, 0.073	0.012	0.031	−0.049, 0.073
Pre-university	0.043	0.030	−0.016, 0.102	0.024	0.030	−0.034, 0.083	0.024	0.030	−0.034, 0.083
University	**0.061**	0.030	0.002, 0.121	0.022	0.031	−0.038, 0.082	0.021	0.031	−0.039, 0.081
Others	Ref			Ref			Ref		
*Income*									
Less than RM 2500	**−0.031**	0.010	−0.051, −0.012	−0.014	0.011	−0.034, 0.007	−0.014	0.011	−0.034, 0.007
RM 2500–RM 3170	−0.008	0.013	−0.033, 0.017	−0.004	0.012	−0.028, 0.021	−0.003	0.013	−0.028, 0.021
RM 3171–RM 3970	−0.019	0.015	−0.047, 0.010	−0.013	0.014	−0.041, 0.015	−0.013	0.014	−0.041, 0.015
RM 3971–RM 4850	Ref			Ref			Ref		
*Financial strain*									
Not enough	**−0.057**	0.011	−0.079, −0.036	**−0.046**	0.011	−0.068, −0.024	**−0.046**	0.011	−0.067, −0.024
Enough for basic needs	−0.012	0.007	−0.026, 0.002	−0.007	0.007	−0.021, 0.007	−0.007	0.007	−0.021, 0.007
Enough to buy all the necessities				Ref			Ref		
*Have a skills certificate*									
Ya	−0.005	0.011	−0.027, 0.018	0.007	0.011	−0.015, 0.029	0.007	0.011	−0.015, 0.029
Tidak	Ref			Ref			Ref		
*Attitude towards skills education*									
Negative (<16)	**−0.019**	0.006	−0.031, −0.007	**−0.013**	0.006	−0.025, −0.001	−0.011	0.007	−0.024, 0.002
Positive (17–23)	Ref			Ref			Ref		
*Knowledge towards skills education*									
Poor (<11)	**−0.015**	0.007	−0.029, −0.001				−0.007	0.008	−0.022, 0.008
Good (12–16)	Ref						Ref		

The bold values mean significant at *p* < 0.05.

## 4 Discussion

In this study, we examined socio-demographic, knowledge, and attitude towards skills education factors associated with HRQOL among Malaysian low-income populations during the COVID-19 pandemic. The WHO recommended preventive measures to flatten the curve by breaking the infection chain, including infection control and social distancing measures (39). Following the surge in new daily cases, Malaysia’s Prime Minister issued a Movement Control Order (MCO) on 18 March 2020. These lockdowns and social distancing measures have had profound negative implications on the population’s mental health and the country’s economy (14, 40). In this section, we discuss the results of our analyses and consider implications for policy, practice, and future research.

The analyses highlight the knowledge and attitude towards skills education, the HRQOL level among them, and the factors most associated with HRQOL. With a sample of 1,997 low-income households in Malaysia, the results demonstrated that most respondents had good knowledge of skills education. In contrast, more than half had a positive attitude towards skilled education. The EQ-5D-5L score was 0.94 (SD 0.15). This value reflects the HRQOL of the study of the low-income Malaysian adult population. There is a significant, negative relationship between age and HRQOL. However, those with financial constraints and a negative attitude towards skills education suffer from a lower quality of life. However, additional adjustment for knowledge and attitude towards skills education was no longer significantly associated with HRQOL, suggesting that knowledge towards skills education accounted for the relationship between attitude towards skills education and HRQOL.

Countries use different value standards and instruments to assess the population’s HRQOL (41). Research has shown that the EQ-5D-5L has acceptable construct validity in healthy or vulnerable Malaysians (35, 37). The recorded EQ-5D-5L value was higher than that of a previous study conducted in 2016 among a low socioeconomic population by Wan Puteh et al. (25). The smaller sample size of the study included disabled people and Orang Asli. This is due to various low-income populations’ demographic, social, and environmental factors that made the quality of life more complex to explore and discuss. It is recommended that future research improve the recruitment of people by including those with this vulnerable condition, as their views on knowledge and attitudes towards skill education may be different.

Ageing involves physiologic changes and a progressive decline of an individual’s functional organs, which can affect health and wellbeing, subsequently affecting the quality of life (42, 43). Our study found a negative correlation between older age and lower HRQOL. This concurs with a previously conducted study (4, 44). In addition, our study found that while more than half of respondents had enough money to buy necessities, 12.1% faced financial constraints limiting them from purchasing basic needs. Similarly, an individual’s financial status was associated with HRQOL in the regression model, with those with financial constraints having lower HRQOL (45).

Countries use different value standards and instruments to assess the population’s HRQOL (46). However, research has shown that the EQ-5D-5L has acceptable construct validity in healthy or vulnerable Malaysians (27, 29). The recorded EQ-5D-5L value was higher than that of a previous study with a smaller sample size conducted in 2016 among a low-socioeconomic population, including disabled people and Orang Asli by Wan Puteh et al. (25). Meanwhile, a random household survey in rural China reported that the HRQOL of individuals living in poverty is lower than that of the general population (45). In another nationally representative survey conducted in Turkey, lower aged, female, and less educated are the predictors of lower quality of life (4). Various demographic, social, and environmental factors of low-income populations that made the quality of life more complex to explore and discuss. Nevertheless, identifying factors that can improve the HRQOL can indicate the need for integrated policies and specific decision-making, including promoting the involvement of the low-income population in skilled training.

Malaysia has set out to achieve zero hardcore poverty by December 2025, a decent standard of living for all Malaysians regardless of sex, ethnicity, or location, and reduced inequalities among their people under the Twelfth Malaysia Plan 2021 to 2025 (12MP) (29). Furthermore, Malaysia recognises the importance of Technical and Vocational Education and Training (TVET) in industry and lifelong learning for skill enhancement (46). Thus, the Malaysian government allocated RM6.6 billion in the 2022 Budget to empower TVET through various initiatives to be carried out by relevant ministries and agencies under the 12th Malaysia Plan (47).

Along with the development of the industrial revolution 4.0 (IR 4.0), the skills possessed by employees should match the skills desired by the industry. Our study found that only 7.5% of respondents had skilled education. As skilled education is critical to Malaysia’s aspirations to become a developed nation and to achieve the goal set in the 12MP, the Malaysian government was committed to five core thrusts in transforming TVET, including training 300,000 Malaysians from the lower income or B40 group from 2019 until 2025 (48). TVET will provide a platform for people to learn the principles of technology and human capital development, particularly in developing skills and the workforce through extensive educational knowledge, followed by a more specific skill training process through various programmes offered by this institution (49). As a result, employment opportunities for skilled workers will be more secure, and high-income careers can be realised.

Previous study investigated factors influencing healthcare outcomes population, revealing associations with labour status, self-perceived health, and specific health conditions (41). Taking an example from nursing education, a positive practice environment, particularly factors like quality outcomes and managerial support, significantly impacted nurse educator outcomes (50, 51). Additionally, the earlier study examined acceptance of medical treatment, revealing that factors such as educational information, economic status, health knowledge, and physical health played a role in acceptance (52). In summary, the previous study offered valuable insights across diverse domains, highlighting the intricate interplay of various determinants, including education, and their impact on health outcomes in low-income populations. This phenomenon could be further explored in the TVET sector, where an increase in technical education for low-income individuals may enhance their health literacy and outcomes. Consequently, there is a pressing need to address recruitment and retention in this area in the future.

A declaration among UN Member nation’s strategy to end poverty has multisectoral involvement that highlights Education for all (EFA); TVET for All and Information and communication technologies (ICT) are the keys to empowering people (53). A study conducted in Malaysia found that community enhancement training skill programmes successfully improved the participants’ skills and quality of life (54). At the same time, studies conducted in different countries showed that TVET is among the principal drivers for sustainability, progress, poverty alleviation, and improved quality of life (55, 56). In addition, a review of the literature on global practices of TVET summarised that TVETs play a key role in enabling nations to solve issues such as poverty, gender equity and equality, a healthy society, and a peaceful society (57). Thus, the involvement of the lower income population in TVET can elevate their working skills, eventually increasing their income and quality of life.

Knowledge helps improve attitude and promote positive behaviour and the cue for action, as reported by the previous study (58, 59). Furthermore, our bivariate analysis found that people with negative attitudes towards skills education have a lower quality of life. As a result, research into the low-income population’s understanding and behaviour is critical to ensuring that they participate in this TVET programme to improve their income and, ultimately, their quality of life. More rigorous efforts should be exerted to reach out to the B40 group with information on how they can up-skill themselves or enrol their children in skill education. This is in line with the policy of making TVET a mainstream education and policy for empowering the B40 group (60).

### 4.1 Limitations

This study yielded a large sample size of community-level respondents at a specific point in the COVID-19 pandemic. A reliable sampling method and a large sample size allow for a comprehensive representation of HRQOL among low-income households. However, based on the initial DOSM sampling frame, a low response from other ethnic groups reduces generalisability among Malaysia’s ethnically diverse population. In addition, the sampling frame was targeted at heads of households which were typically males. As a result, it should be interpreted with caution, especially among females and non-Malays. As a result, it should be interpreted with caution, especially among non-Malays. Second, the length of the data collection period changed. Thus, changes in QOL may have slowed as time passed due to factors such as rising COVID-19 cases, which resulted in lower economic output. Finally, a cross-sectional study design was unable to establish the cause-and-effect relationship between the factors and HRQOL. Eventually, while the small number of missing data may impact the study analysis, using a self-reported questionnaire may result in reporting bias in this study. Despite these limitations, the study adds to the growing body of literature that recognises the importance of low-income populations’ knowledge and attitudes towards skilled education, which affects their health-related quality of life.

## 5 Conclusion

Our study identified associations between socio-demographics, knowledge, and attitudes towards skilled education with HRQOL. Following the impact of the COVID-19 pandemic, the Malaysian government acknowledged the importance of improving the economy of the low-income population, as they were the most affected populations following the unprecedented economic crisis. Therefore, the low-income group needs to be made aware that they can meet the needs of today’s industry through their skills education. In addition, improving the family economy will reduce financial strain, increasing HRQOL. So, it is recommended to either conduct a prospective study or a qualitative study. Prospective studies will affirm a causal relationship. At the same time, in-depth information can be gathered using a qualitative approach to address the barrier to obtaining skilled education. The findings will give policymakers strategies to improve low-income people’s participation in skill education and raise everyone’s quality of life in Malaysia.

## Data availability statement

The original contributions presented in the study are included in the article/Supplementary material, further inquiries can be directed to the corresponding author.

## Ethics statement

The studies involving humans were approved by University of Malaya Research Ethics Committee (UMREC). The studies were conducted in accordance with the local legislation and institutional requirements. The participants provided their written informed consent to participate in this study.

## Author contributions

RY, MS, HM, RI, and TS conceptualised the study. RY, MS, MT, HM, RI, and TS performed material preparation and data collection. RY, MS, AM, MT, HR, HM, RI, and TS validated data. MS, AM, MT, HR, NT, and MI conducted the formal analysis. The first draft of the manuscript was written by MI, NT, MS, HR, MT, and AM, and all authors commented on previous versions of the manuscript. All authors read and approved the final version of the manuscript.
