# Supplementary material for: TVET programme and health-related quality of life among low-income populations during the COVID-19 pandemic in Malaysia
Source: Front Public Health. 2024 Mar 5;12:1164056. doi: 10.3389/fpubh.2024.1164056 (PMC10948447; doi:10.3389/fpubh.2024.1164056)
Supplement: Supplementary file 1 [file Table_1.DOCX]

TVET program and health-related quality of life among low-income populations during the COVID-19 pandemic in Malaysia

Ruhizan Mohammad Yasin*, Tan Maw Pin, Mas Ayu Said, Mohd Sattar Rasul, Nithiah Thangiah, Hussein Mohammad Rizal, Amirah Shazana Magli, Muslimah Ithnin, Hazreen Abdul Majid, Rozmi Ismail, Tin Tin Su

*** Correspondence:** Ruhizan Mohammad Yasin: [ruhizan@ukm.edu.my](mailto:ruhizan@ukm.edu.my)

**Supplementary Table 1.** The association of EQ-5D-5L scores with participants’ knowledge and attitude towards skill education (n=1997)

| Variables | Mean (SD) | Min-Max | Spearman’s r | *p*-value |
| --- | --- | --- | --- | --- |
| Age | 45.17 (14.113) | 18-91 | -0.177 | 0.001* |
| Household income during COVID-19 (RM) | 2124.54 (1355.10) | 0-4850 | 0.082 | 0.001* |
| Knowledge of skill education | 11.98 (4.10) | 4-16 | 0.047 | 0.038 |
| Attitudes towards skills education | 16.23 (3.92) | 6-23 | 0.057 | 0.011* |

**p*<0.05

**Supplementary Table 2.** Soal selidik pengetahuan mengenai Pendidikan dan Latihan Teknikal dan Vokasional (Versi Melayu)/ Knowledge towards Technical and Vocational Education and Training (TVET) questionnaire (Malay version).

*Based on the scale below, please mark the answer that describes your situation.*

| Items | Scale | | | |
| --- | --- | --- | --- | --- |
|  | Strongly disagree | Disagree | Agree | Strongly agree |
| Saya mempunyai pengetahuan dalam mencari peluang untuk meningkatkan pengetahuan / pendidikan.  *I had some knowledge in finding opportunities to improve knowledge /education.* |  |  |  |  |
| Saya mempunyai pengetahuan tentang bantuan dari kerajaan untuk meningkatkan pendidikan dan latihan.  *I know assistance from the government to improve education and training.* |  |  |  |  |
| Saya mempunyai pengetahuan dalam mencari peluang dan bantuan pendidikan daripada agensi lain daripada kerajaan (cth. NGO & Industri).  *I have knowledge in seeking educational opportunities and assistance from agencies other than the government (e.g., NGOs & Industry).* |  |  |  |  |
| Saya tahu apakah kemudahan pendidikan yang disediakan oleh kerajaan.  *I know what educational facilities to improve skills provided by the government.* |  |  |  |  |

**Supplementary Table 3.** Soal selidik sikap mengenai Pendidikan dan Latihan Teknikal dan Vokasional (Versi Melayu)/ Attitude towards Technical and Vocational Education and Training (TVET) questionnaire (Malay version).

*Based on the scale below, please mark the answer that describes your situation.*

| Items | Scale | | | |
| --- | --- | --- | --- | --- |
|  | Strongly disagree | Disagree | Agree | Strongly agree |
| Saya tidak meneruskan pelajaran kerana masalah kewangan.  *I did not continue my studies due to financial problems* |  |  |  |  |
| Saya percaya bahawa sentiasa terdapat penyelesaian kepada sebarang cabaran.  *I believe that there is always a solution to any challenge.* |  |  |  |  |
| Pandemik COVID-19 memberi kesan negatif kepada kestabilan sosial dan emosi saya.  *I have negative feelings and emotional instability due to the COVID-19 pandemic* |  |  |  |  |
| Penting untuk keluarga saya sentiasa mencari peluang untuk meningkatkan kemahiran pekerjaan.  *I believe it is essential to look for opportunities to improve job skills.* |  |  |  |  |
| Saya tahu tentang pusat-pusat pentauliahan kemahiran yang berdekatan.  *I knew about skills accreditation centres near me.* |  |  |  |  |
| Selepas pandemik COVID-19, saya percaya kami perlu meningkatkan kemahiran vokasional untuk membantu keluarga kami.  *I believe that after the COVID-19 pandemic, there is a need to improve vocational skills to help the family.* |  |  |  |  |

**Supplementary Table 4.** EQ-5D-5L (Malay Version)

*Under each heading, please tick (/) one box that best describes your health today.*

| PERGERAKAN / MOBILITY |  |
| --- | --- |
| Saya tidak menghadapi masalah untuk berjalan  *I have no problems in walking about* |  |
| Saya menghadapi sedikit masalah untuk berjalan  *I have slight problems in walking about* |  |
| Saya menghadapi masalah yang sederhana untuk berjalan  *I have moderate problems in walking about* |  |
| Saya menghadapi masalah yang teruk untuk berjalan  *I have severe problems in walking about* |  |
| Saya tidak berupaya untuk berjalan  *I am unable to walk about* |  |
| PENJAGAAN DIRI / SELF-CARE |  |
| Saya tidak menghadapi masalah untuk membersihkan diri atau memakai sendiri pakaian saya  *I have no problems washing or dressing myself* |  |
| Saya menghadapi sedikit masalah untuk membersihkan diri atau memakai sendiri pakaian saya  *I have slight problems washing or dressing myself* |  |
| Saya menghadapi masalah yang sederhana untuk membersihkan diri atau memakai sendiri pakaian saya  *I have some problems washing or dressing myself* |  |
| Saya menghadapi masalah yang teruk untuk membersihkan diri atau memakai sendiri pakaian saya  *I have severe problems washing or dressing myself* |  |
| Saya tidak berupaya untuk membersihkan diri atau memakai sendiri pakaian saya  *I am unable to wash or dress myself* |  |
| AKTIVITI-AKTIVITI BIASA (misalnya bekerja, belajar, membuat kerja rumah, aktiviti-aktiviti keluarga atau masa lapang) /  USUAL ACTIVITIES (e.g. work, study, housework, family or leisure activities) | |
| Saya tidak menghadapi masalah untuk melakukan aktiviti-aktiviti biasa saya  *I have no problems doing my usual activities* |  |
| Saya menghadap sedikit masalah untuk melakukan aktiviti-aktiviti biasa saya  *I have slight problems doing my usual activities* |  |
| Saya menghadapi masalah yang sederhana untuk melakukan aktiviti-aktiviti biasa saya  *I have some problems doing my usual activities* |  |
| Saya menghadapi masalah yang teruk untuk melakukan aktiviti-aktiviti biasa saya  *I have severe problems doing my usual activities* |  |
| Saya tidak berupaya untuk melakukan aktiviti-aktiviti biasa saya  *I am unable to do my usual activities* |  |
| KESAKITAN / KETIDAKSELESAAN / PAIN / DISCOMFORT |  |
| Saya tidak berasa sakit atau tidak selesa  *I have no pain or discomfort* |  |
| Saya berasa sakit atau tidak selesa sedikit  *I have slight pain or discomfort* |  |
| Saya berasa sakit atau tidak selesa yang sederhana  *I have moderate pain or discomfort* |  |
| Saya berasa sakit atau tidak selesa yang teruk  *I have severe pain or discomfort* |  |
| Saya berasa sakit atau tidak selesa yang teramat sangat  *I have extreme pain or discomfort* |  |
| ANXIETY / DEPRESSION |  |
| Saya tidak berasa risau atau murung  *I am not anxious or depressed* |  |
| Saya berasa risau atau murung sedikit  *I am slightly anxious or depressed* |  |
| Saya berasa risau atau murung yang sederhana  *I am moderately anxious or depressed* |  |
| Saya berasa risau atau murung yang teruk  *I am severely anxious or depressed* |  |
| Saya berasa risau atau murung yang teramat sangat  *I am extremely anxious or depressed* |  |
